# Ensemble Neuroevolution-Based Approach for Multivariate Time Series Anomaly Detection

**DOI:** 10.3390/e23111466

**Published:** 2021-11-06

**Authors:** Kamil Faber, Marcin Pietron, Dominik Zurek

**Affiliations:** Department of Computer Science, AGH University of Science and Technology, Adama Mickiewicza 30, 30-059 Krakow, Poland; kfaber@agh.edu.pl (K.F.); dzurek@agh.edu.pl (D.Z.)

**Keywords:** neuroevolution, anomaly detection, ensemble model, CNN, time series, deep learning

## Abstract

Multivariate time series anomaly detection is a widespread problem in the field of failure prevention. Fast prevention means lower repair costs and losses. The amount of sensors in novel industry systems makes the anomaly detection process quite difficult for humans. Algorithms that automate the process of detecting anomalies are crucial in modern failure prevention systems. Therefore, many machine learning models have been designed to address this problem. Mostly, they are autoencoder-based architectures with some generative adversarial elements. This work shows a framework that incorporates neuroevolution methods to boost the anomaly detection scores of new and already known models. The presented approach adapts evolution strategies for evolving an ensemble model, in which every single model works on a subgroup of data sensors. The next goal of neuroevolution is to optimize the architecture and hyperparameters such as the window size, the number of layers, and the layer depths. The proposed framework shows that it is possible to boost most anomaly detection deep learning models in a reasonable time and a fully automated mode. We ran tests on the SWAT and WADI datasets. To the best of our knowledge, this is the first approach in which an ensemble deep learning anomaly detection model is built in a fully automatic way using a neuroevolution strategy.

## 1. Introduction

In the anomaly detection field, deep learning models achieve the best results on well-known benchmarks. These are mainly deep autoencoders based on Long-Short Term Memory (LSTM) layers, convolutional layers, and a fully connected sequence of layers. A wide variety of autoencoders are used, such as variational, denoising, and adversarial autoencoders. Research shows that further improvements, such as adding a discriminator as an additional verification module or other Generative Adversarial Network (GAN) based autoencoder modifications, can boost the detection results. Recently, promising results using deep graph neural networks in anomaly detection have also been shown [1].

Neuroevolution is a form of artificial intelligence that uses evolutionary algorithms to generate artificial neural networks (ANNs), parameters, topologies, and rules. The most popular algorithms are NEAT, HyperNEAT, and coDeepNEAT [2]. In the presented approach, neuroevolution is used to generate an optimal ensemble anomaly detection model.

In this paper, we propose a high-level ensemble approach fine tuned by a neuroevolution algorithm. The presented method is model independent and can be adapted to any deep learning anomaly detection model. The main advantage of the algorithm is its fully automated mode. The novelty of the proposed algorithm is that we added new search dimensions, including the training data distribution, dividing data into subgroups, and searching for the optimal composition of the ensemble model.

The proposed neuroevolution search space is based on creating encoders and decoders from single neural layers, such as fully connected, convolutional, recurrent, and attention layers. There are two main dimensions of optimization. Therefore, two populations are inside the algorithm. The first is the population of the models from which genetic operators evolve new generations of models. The second is the subgroup population, which is needed to form the ensemble model from the models’ population. This work concentrates on the data optimization stage and the setting up of the ensemble model. It shows how this aspect can improve nonensemble models. The last step in the neural architecture search (NAS) is fitness definition. Fitness is the sum of losses from the training dataset and the random reduced validation dataset in the presented approach.

The main advantages of the presented algorithm are that it enables building the ensemble model in automatic mode and creates a vast search space between various deep learning autoencoders, GAN architectures, and optimal training data subgroups.

## 2. Related Works

Anomaly detection is a popular research subject. The basic unsupervised methods include linear-model-based methods [3], distance-based methods [4,5], density-based methods [6], isolation-based methods [7], and many others. The best f1-score for these methods is 23% on the SWAT and 9% on the WADI datasets. However, deep learning methods have recently gained significant improvements in anomaly detection over the aforementioned approaches. The most popular deep learning models for multivariate anomaly detection are autoencoder (AE) models, which use the reconstruction error for the anomaly inspection. Zong et al. proposed a deep autoencoding Gaussian mixture model (DAGMM) [8] that jointly optimizes the deep autoencoder parameters and the mixture model simultaneously. This solution yields an f1-score of 55% for the SWAT and 20% for the WADI datasets. Park et al. introduced the LSTM-VAE model [9], replacing the feedforward network in the variational autoencoder (VAE) with LSTM. As a result of this approach, it was possible to gain an f1-score of 75% for the SWAT and 25% for WADI the datasets. Russo et al. used an autoencoder that consists of 1D convolution layers [10]. This model was tested with the *Urban Water Observatory Initiative* (www.eawag.ch/uwo, accessed on 1 November 2021) datasets and has an anomaly detection accuracy of 35%. Audibert et al. proposed a fast and stable method called USAD [11], which is based on adversely trained autoencoders. This model contains only fully connected layers and achieves a 79% detection anomaly for the SWAT and 23% for the WADI dataset. Generative adversarial networks (GANs) as anomaly detectors were proposed in [12]. The authors applied LSTM as the generator and discriminator models in the GAN framework and used a combination of both model errors (DR-score) to detect anomalies. The anomaly accuracy for this model is 77% for the SWAT and 37% for the WADI datasets. Deng et al. [1] achieved an f1-score of 81% for the SWAT and 57% for the WADI datasets through the use of a graph neural network (GNN). The mentioned deep learning models, LSTM, USAD, and CNN 1D, are the baseline for the solutions proposed in this paper. Our preliminary observations showed that a single model (autoencoder) very often cannot handle many input signals efficiently. The hypothesis is that the ensemble model (especially that based on bagging) can improve anomaly detection in such cases. The next aspect is that the architectures of the models presented in the mentioned papers have not been sufficiently explored. The search for optimal architectures in other fields such as image classification showed that this approach could explore more possibilities and outperform models designed by humans. Among the search criteria was speed. Therefore, the neuroevolution technique was chosen to perform a search in addition to the reinforcement learning approach.

Recently, neuroevolution algorithms have been used in many machine learning tasks to improve the accuracy of deep learning models [13]. In [14], neuroevolution search was used to evolve neural networks for object classification in high-resolution remote sensing images. In [15], the authors presented a neuroevolution algorithm for standard image classification. The authors in [2] showed the neuroevolution strategy scheme for language modeling, image classification, and object detection tasks. This was based on the co-evolutionary NEAT algorithm, which has the two following levels of optimization: the first one is single deep learning subblock optimization; the second one is a composition of subblocks to form a whole network. The presented results showed that optimized models achieved better results than those of models designed by humans in most cases.

## 3. Autoencoder Architecture

Autoencoders are unsupervised learning models in which the neural network is trained to learn the compressed representation of raw data. These models consist of two parts: an encoder *E* and a decoder *D*. The encoder learns how to efficiently compress and encode the input data *X* to represent them in reduced dimensionality—latent variables *Z*. The decoder is taught how to reconstruct the latent variables *Z* back to their original shape. The model is trained to minimize *reconstruction loss*, which means reducing the difference between the output of the decoder and the original input data; this can be expressed as:

(1) $$L\left(X,\hat{X}\right)=||X-AE\left(X\right){\left|\right|}_{2}$$

where:

(2) AE(X)=D(Z),Z=E(X)

The most straightforward kind of autoencoder is an *undercomplete autoencoder (UAE)*. These models learn the most essential and relevant attributes of the input data by using a bottleneck with a smaller dimension than the input data. Another type of autoencoder, called the *denoising autoencoder (DAE)*, extracts essential features from the data by reconstructing the original input after it has been contaminated by noise. In unsupervised tasks, the most popular type of autoencoder is the *variational autoencoder (VAE)*. This type of autoencoder replaces the bottleneck vector with two vectors: one representing the mean of the distribution and the second representing the standard deviation of the distribution. The VAE for a given input in the encoding phase determines the distribution of the latent variables. By contrast, the decoder determines the distribution of the inputs corresponding to the given latent variables. Autoencoders are widely used in many fields, such as online intrusion detection [16], malware detection [17], and anomaly detection in streaming data [18]. This model can consist of various layers, e.g., fully connected layers, CNN, and LSTM.

## 4. Neuroevolution Ensemble Approach

**Figure 1:**
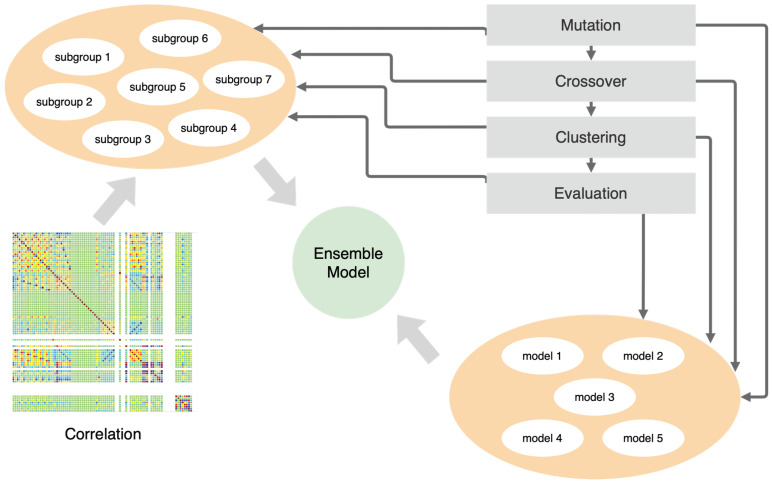
The architecture of the framework.

The prototype of our framework presented in Figure 1 consists of two separate populations. The first is the models’ population, and the second is the data subgroup population. Equation (3) describes the definition of the model population where θ is a weight tensor. Each model is a sequence of layers of encoders and decoders (Equations (4) and (5)) that describes the input multivariate signal. The framework enables the formation of the ensemble model using an approach similar to the bagging-based technique. The whole ensemble model is a set of submodels working on subgroups of the input signals (Equations (6) and (7)). The ensemble models form a population set (Equation (9)).

(3) $$P_{M}=\left\{F_{\Theta }^{i},\Theta =\left\{\theta_{0},\theta_{1},\ldots ,\theta_{N}\right\}\wedge i\in \left\{1,2,\ldots ,population\_size\right\}\right\}$$

(4) $$F_{\Theta }^{i}\left(X\right)=f_{\theta_{N}}^{i}\left(f_{\theta_{N-1}}^{i}\ldots \left(f_{\theta_{0}}^{i}\left(X\right)\right)\right)$$

(5) $$X=\{x_{i},i\in \left\{1,2,\ldots ,nr\_of\_sensors\right\}\}$$

The framework starts with generating initial groups of input signals using correlation (this is explained in detail in the subsections below); then, it mutates models and groups via the genetic algorithm. In parallel, the genetic operators optimize the single models in each subgroup by making changes in the topology of the neural models (e.g., the length of the model and layer parameters). The final effect of these actions is an ensemble model optimized to detect anomalies. The ensemble model is defined as follows (where M=ensemble_size):

(6) $$F_{ensemble}^{i}\left(X\right)\Leftrightarrow \left(F_{\Theta }^{i0}\left(x_{i0}\right),F_{\Theta }^{i1}\left(x_{i1}\right),\ldots ,F_{\Theta }^{iM}\left(x_{iM}\right)\right)$$

(7) $$x_{i0}\wedge x_{i1}\ldots \wedge x_{iM}\subset X$$

(8) $$|x_{ij}|\leq nr\_of\_sensors,\forall j\in \left\{1,2,\ldots ,M\right\}$$

(9) $$P_{E}=\left\{F_{ensemble}^{i},i\in \left\{1,2,\ldots ,ensemble\_population\_size\right\}\right\}$$

### 4.1. General Schema of the Proposed Solution

During our experiments with various models, we noticed that almost all models detect a similar set of anomalies despite changes in their hyperparameters. Of course, the results differed depending on the hyperparameters, but none of the changes significantly impacted the detection. Therefore, we decided to apply an ensemble model based on dividing available input signals into smaller groups and training each model on a separate subset of signals. As a result of this, the models could discover more specific dependencies and relations between signals. We present a simplified schema of our approach in Algorithm 1. Additionally, models inside each subgroup are optimized by changing their internal architectures (Algorithms 2–4). The evolution of the single model is conducted in four main steps in a loop (Algorithm 2): clustering by model length, crossover, mutation, and choosing the best models in clusters. Algorithms 3 and 4 contain detailed descriptions of the crossover and mutation. We define the crossover as a function that has two parent models and an id of the layer as input parameters (Equation (10)). It generates two new children models (Equation (11)).

(10) c:Parents×layer_id→Children

(11) $$c\left(F_{\Theta }^{i},F_{\Theta }^{j},l\_id\right)\to \left(F_{\Theta }^{i^{\prime }},F_{\Theta }^{j^{\prime }}\right)$$

(12) $$f_{\Theta l}^{i^{\prime }}=f_{\Theta l}^{j}$$

(13) $$f_{\Theta l}^{j^{\prime }}=f_{\Theta l}^{i}$$

The crossover can exchange between parents’ activation functions, convolutional, LSTM, or fully connected layers (Equations (12) and (13)). The last option is to exchange the hyperparameters between parents, such as the learning rate and weight decay. We define mutation as a function with a model, layer id, and layer features to be mutated as input parameters (Equation (14)).   

(14) m:Parent×layer_id×layer_feature→Child

(15) $$m\left(F_{\Theta }^{i},l\_id,l\_feature\right)\to F_{\Theta }^{i^{\prime }}$$

In this case, the feature and layer_id determine what the mutation operator will do. They can change the activation function or parameters of the convolutional, LSTM, or fully connected layers (e.g., depth and kernel_size). The other option is to change the length of the model (adding or removing a layer) or update the hyperparameters. The result is a new modified model (Equation (15)).

Then, the neuroevolution approach is applied to search for an optimal partition into groups (Line 1, Algorithm 1). After classifying data points using every model (Line 2, Algorithm 1), we used a voting mechanism to determine whether a data point should be considered as an anomaly by the whole ensemble model (Line 3, Algorithm 1).
**Algorithm 1:** Simplified general schema of our approach. **Result:** Classification of the anomalies**1** Find the best partition of signals into groups using a genetic algorithm (Algorithm 5);**2** Find the best model for a group (Algorithm 2);**3** Train and evaluate a separate model for every group (Algorithm 6);**4** Evaluate an ensemble model using a threshold voting algorithm in which every group of signals has the same voting power
**Algorithm 2:** Model evolution.
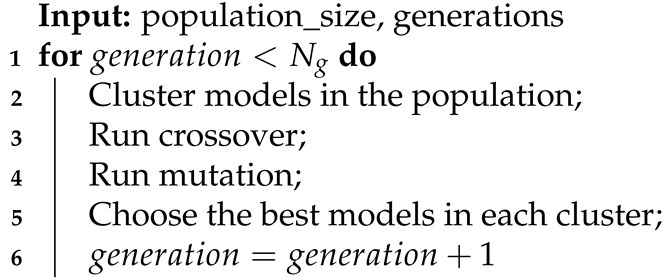

**Algorithm 3:** Single model crossover. **Input:** parent_1, parent_2**1** Choose the feature that will be exchanged between parents;**2** If the feature is activation_ function, then exchange_activation;**3** If the feature is layer, then exchange_layer;**4** If the feature is change_length, then remove_or_add_layer;**5** If the feature is hyperparameter, then exchange_opt;

To find an optimal partition of input signals into groups, we applied a genetic algorithm. Algorithm 5 presents a simplified schema of the genetic algorithms.

The single gene provides information showing that a feature *f* is present in a group *t*. The single solution represents *k* groups, each containing zero or more input signals. A sample solution for k=3 could be: [[0,1,5,12],[2,3,4,9],[6,7,9]], where numbers in groups indicate which input signals are present. Population *P* contains $N_{P}$ solutions.

The parameters for the neuroevolution approach in this work are as follows:

- *k*—maximal number of submodels in an ensemble model;
- $p_{m}$—probability of the mutation in a single group of input signals;
- $N_{g}$—number of generations in a genetic algorithm;
- $N_{P}$—size of the population in a genetic algorithm;
- $N_{par}$—number of parents mating;
- $N_{ep}$—number of epochs to train while calculating fitness.

**Algorithm 4:** Single model mutation **Input:** solution**1** Choose a feature;**2** If the feature is activation_function, then change_activation;**3** If the feature is layer, then change_layer_parameters;**4** If the feature is hyperparameter, then change_opt;
**Algorithm 5:** Genetic algorithm.
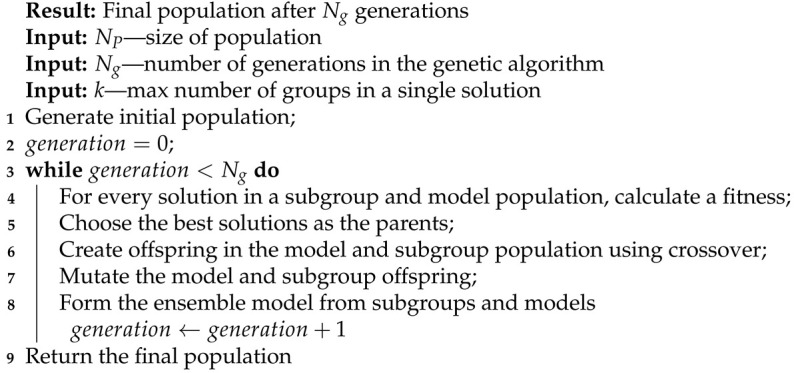


### 4.2. Genetic Algorithm for Building the Ensemble Model

To improve the convergence of the genetic algorithm, we created it based on the correlation between input signals instead of using a random initial population. We used hierarchical clustering with the addition of some randomness to achieve a diverse population.

Algorithm 6 presents a method for calculating the fitness for a single solution. For each dataset used (SWAT and WADI), we split a normal part of the data into training and validation datasets. We calculated the fitness for every feature group in the solution. As the first step, we trained a chosen model on selected input signals from the training data for a given number of epochs (Line 3, Algorithm 6). After that, we evaluated the trained model on training and validation data (Lines 4 and 5, Algorithm 6), calculating the losses. To normalize the loss, we calculated the weighted loss from the training and validation datasets (Lines 6 and 7), and we also divided the weighted loss by the number of input signals in the group (Line 8, Algorithm 6). The final fitness for every solution is a negated sum of losses for groups in the solution (Lines 9 and 10, Algorithm 6). The value is negated because we wanted to minimize the total loss of an ensemble model, while in the genetic algorithm, the goal is to maximize the fitness.

During the crossover part (Line 6, Algorithm 5), we created a new solution based on two selected parents (Equation (16). Algorithm 7 presents the detailed steps of the crossover. For every pair of groups of parents, we determined which range of input signals is present in the groups and chose the random split point (Lines 3–5, Algorithm 7). The new group of offspring then contains parts of the groups from both parents (Lines 6–13, Algorithm 7 and Equations (17) and (18)).
**Algorithm 6:** Fitness calculation.
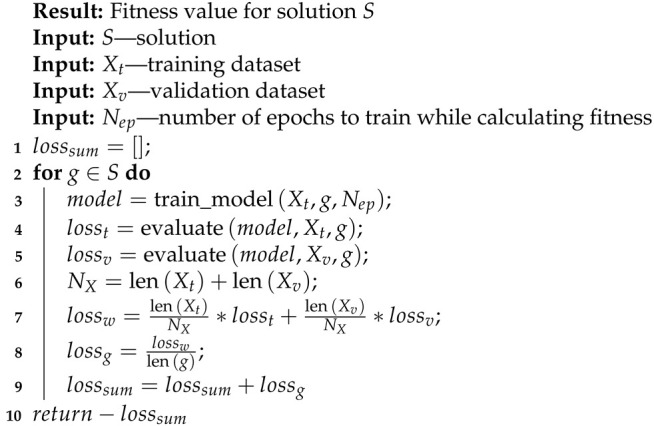

**Algorithm 7:** Crossover algorithm.
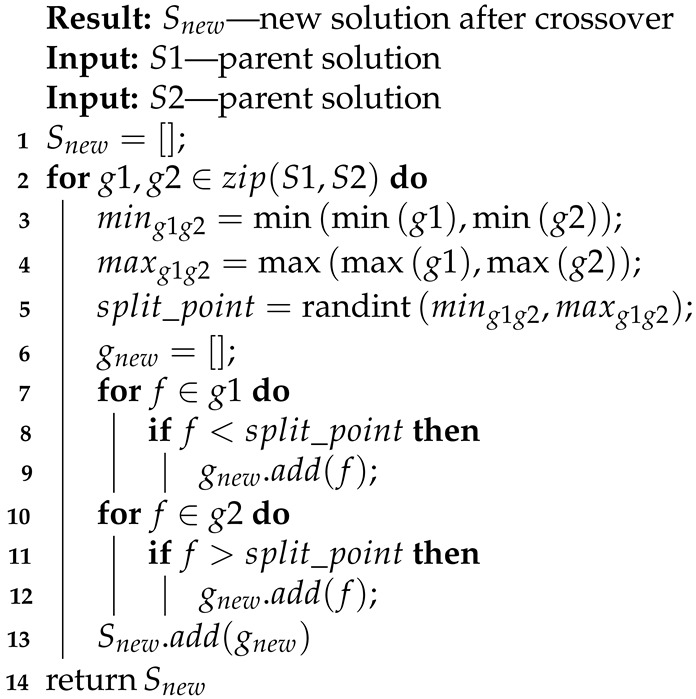


Offspring created via a crossover algorithm can also be affected by mutations (Line 7, Algorithm 5).

(16) $$c_{e}:Parents\times probability\to Children$$

(17) $$c_{e}\mapsto \left(c_{g}\left(i0,j0,split0\right),c_{g}\left(i1,j1,split1\right),\ldots ,c_{g}\left(iM,jM,splitM\right)\right)$$

(18) $$c_{g}:x_{iN}\times x_{jN}\times split\_point\to \left[x_{iN}\left[0\right],\ldots ,x_{iN}\left[split\_point\right],\ldots ,x_{jN}\left[-1\right]\right]$$

In our work, we used three types of mutation for ensemble models (Equations (19) and (20)):

1. Duplicating a selected feature in another group (presented in Algorithm 8), Equation (21);
2. Vanishing input signals that exist in more than one group in a single solution (presented in Algorithm 9), Equation (22);
3. Adding input signals that do not exist in any group in a single solution (presented in Algorithm 10), Equation (23).

The goal of mutations is to help maintain diversity in the population. Mutation 1 allows for the same features to be available in a few groups. Mutation 2 protects solutions from having a few groups with the same input signals and from overusing any feature. Mutation 3 makes it possible to restore input signals lost in other genetic operations.

(19) $$m_{e}:Parent\times probability\times type\to Child$$

(20) $$m_{e}\left(i,prob\right)\mapsto \left(m_{e}\left(i0,prob\right),m_{e}\left(i1,prob\right),\ldots ,m_{e}\left(iM,prob\right)\right)$$

(21) $$m_{em}:x_{ik}\times x_{im}\times f\to \left(x_{ik}-f,x_{im}\cup f\right)$$

(22) $$m_{ev}:x_{ik}\times f\to x_{ik}-f$$

(23) $$m_{en}:x_{ik}\times f\to x_{ik}\cup f$$

**Algorithm 8:** Moving mutation.

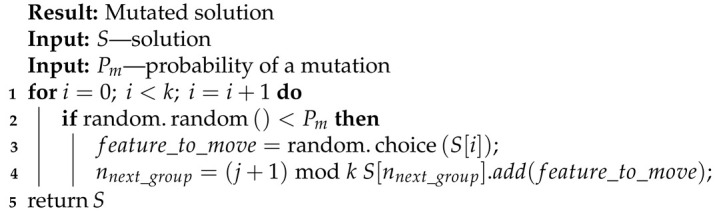



**Algorithm 9:** Vanishing mutation.

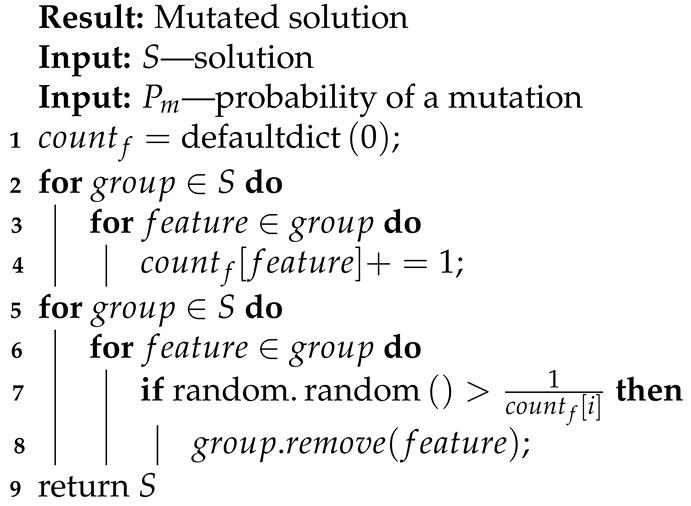



**Algorithm 10:** New input signals mutation.

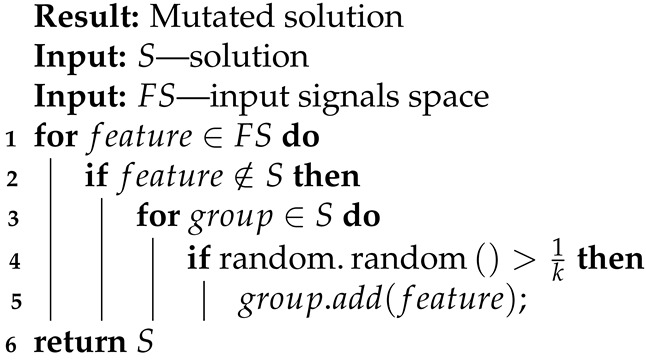



## 5. Results

In this section, we describe the used datasets and models. We also demonstrate the improvements that were possible to achieve by the usage of the proposed solutions. We provide a comparison with methods from the state-of-the-art articles. We ran all the presented calculations on an Nvidia Tesla V100-SXM2-32GB (https://www.nvidia.com/en-us/data-center/v100/, accessed on 1 November 2021). In order to reduce both training times, i.e., during the evolution algorithm and the final training, each subgroup was calculated on a separate GPGPU. The genetic algorithm parameters were the same for all experiments and are presented in Table 1 (the column *basic value*). Moreover, the model that gained the best results (CNN 1D) was also run once again with a higher value of the parameters *population size* and *parents mating* (the column *Rerun value* in Table 1) to determine how this affects the efficiency of the algorithms.

### 5.1. Datasets

We used the following as the training and testing data:

- The *Secure Water Treatment (SWaT) Dataset* [19], which contains data gathered from a scaled-down version of a real water treatment plant. The data were collected for 11 d in two modes—7 d of normal operation of the plant and 4 d during which there were cyber and physical attacks executed;
- The *Water Distribution (WADI) Dataset* [20], which contains data from a scaled-down version of a water distribution network in a city. The collected data contain 14 d of normal operation and 2 d during which there were 15 attacks executed. As presented in Table 2, there are two WADI collections from 2017 and 2019 available. In our experiments, we used the newest version as per the recommendation of the authors of the dataset.

### 5.2. Models

In this paper, in order to detect anomalies, we used three models of the autoencoder. The first of these was proposed in [10], where the encoder contained three 1D CNN layers with kernel sizes $k_{1}=8$, $k_{2}=6$, and $k_{3}=4$ and filter maps $f_{1}=64$, $f_{2}=128$, and $f_{3}=256$. Each CNN layer is followed by the LReLU [21] activation function and batch normalization calculation. The decoder is a mirror reflection of the encoder where transposed CNN layers replace CNN layers. The second model is a variational autoencoder [9] where both the encoder and the decoder contain two LSTM layers with hidden sizes equaling 16, followed by the LReLU activation function. The batch size is thirty-two for the training phase and one for the test phase. The third model is the USAD model proposed in the literature [11]. It utilizes the idea of GANs and the architecture of autoencoders. The USAD model consists of two autoencoders built from one shared encoder and two decoders. They are trained using the proposed two-phase training, including standard autoencoder training and adversarial training specific to GANs.

In the evolution algorithm (see Section 4), each model was trained over 15 epochs, and during the final training, each model was trained over 70 epochs. We divided the multivariate time series into subsequences with a sliding window parameter as an additional parameter for optimization. To speed up the training, we used downsampling with a ratio of five, which reduces the size of the data. As indicated in the literature [11], this operation did not cause a significant drop in accuracy. Table 3 contains the number of trainable parameters for each model used, the optimal values for some of them, and the time that was necessary to perform the whole process. As can be observed, the USAD model contains the highest number of parameters and took the longest time to train.

### 5.3. Experiments

Table 4 contains the collected results from [1,3,4,8,9,11,12]. Additionally, the table contains results produced for this paper. In the case of the WADI dataset, we used the WADI 2019 version (marked as *). Moreover, for our baseline models (USAD, LSTM-VAE, and CNN 1D), we present the outcomes from our experiments for the SWAT dataset. The results were slightly different from the original result, as required to prepare the implementation on our own (marked as **).

Table 5 contains the gained results after splitting the groups through the use of genetic algorithms. Each experiment was run ten times, and the presented result is the best one observed. We can observe a significant improvement on the WADI dataset in the case of the USAD model and CNN-based autoencoder. It had a minor impact on the LSTM-VAE model.

We gained the best results for the CNN 1D autoencoder, and as such, we reran the experiment for this model with a higher value of the following parameters of the genetic algorithm: *population size* and *parents mating*. As a result, it was possible to improve the f1-score by about 2% in the case of the SWAT and WADI datasets. These results are marked as (***) in Table 5. The optimal number of generated subgroups for CNN LSTM-VAE and USAD equals five. For each model, most anomalies were located in two subgroups. We can observe that each proposed autoencoder working on smaller groups of sensors was more sensitive in anomaly detection (a couple of percentage points in the SWAT dataset and over a dozen percentage points in the WADI dataset). Optimal sliding windows already found had sizes of 2 for autoencoders with CNN layers, 4 in the case of LSTM-VAE, and 12 in the case of USAD.

Additionally, by using a single model of neuroevolution, some single base models were improved. The best single model achieved by using the presented algorithm was the CNN 1D-based autoencoder, which had the following parameters: $k_{1}=4$, $k_{2}=2$, $k_{3}=5$, filter maps $f_{1}=50$, $f_{2}=131$, and $f_{3}=197$, as well as sliding windows equaling two (in the base models, the optimal sliding windows is four). The remaining layers were analogous to the basic model. The single model evolution was run in a population with a size equal to 24. After crossover and the mutation operation, the distance function (mainly based on the length of the model) was computed to diversify the offspring population (in addition to the best models, those that were different from each other were also chosen for the next iteration).

Table 6 presents the hyperparameters of the most effective generated CNN 1D model, which on all input sensors (one group) improved the f1-score by about 15% (44% on the WADI dataset, which was the best result gained by a single model). The depth was the same as in the base model, but the hyperparameters changed in the neuroevolution process and, therefore, were better adjusted to the given task. The presented experiments proved that evolution works in two dimensions. It can improve single base models and further improve detection results by generating an ensemble model.

## 6. Conclusions and Future Work

The results showed that the data distribution, dividing the input signals into subgroups, and using an ensemble model can significantly improve the efficiency of the anomaly detection process. The neuroevolution process helps to find near-optimal subgroups. The tests were run on the WADI and SWAT benchmarks. In both cases, the best results were achieved among models other than graph neural network models. The improvements in the WADI dataset were more significant than those in the SWAT dataset, because there are more sensors and samples in the WADI dataset than in the SWAT dataset.

Our paper proved that the neuroevolution approach might have a positive impact on results. Our future work will concentrate on further enhancements of the algorithm. We consider the following the most critical enhancements: an ensemble model based on graph networks; a new crossover to mix different architectures, e.g., attention with a discriminator; graph networks with USAD. We will also run more extended simulations (with more significant populations and more iterations), which can determine the f1-score. 

## Figures and Tables

**Table 1:** Parameters of the genetic algorithm.

Parameter	Basic Value	Rerun Value
Population size	8	16
Number of parents mating	4	8
Mutation probability	0.1	0.1
Number of generations	10	10

**Table 2:** Statistics of the used datasets.

Datasets	# of Input Signals	# of Trainings	# of Tests	# of Anomalies
**SWAT**	51	49,668	44,981	11.97%
**WADI-2017**	123	1,048,571	172,801	5.99%
**WADI-2019**	123	784,571	172,801	5.77%

**Table 3:** Parameters of the models.

Method	Neuroevolution Time on 8 GPGPUs	# of Trainable Parameters	Type of Parameters
**LSTM-VAE**	24 h	2,378,496	sliding windows = 4 #final training epochs = 70 # of epochs during fitness = 15
**USAD**	32 h	3,937,360	sliding windows = 12 #final training epochs = 70 # of epochs during fitness = 15
**CNN 1D**	16 h	366,476	sliding windows = 2, #final training epochs = 70, # of epochs during fitness = 15, learning rate = 0.01

**Table 4:** Anomaly detection accuracy (precision (%), recall (%), f1-score (%)) on two datasets without splitting into groups. Results marked as * were generated by the usage of the WADI-2019 dataset. ** means that we had to reimplement a model on our own.

Method	SWAT			WADI		
	Prec	Rec	f1	Prec	Rec	f1
**PCA**	24.92	21.63	0.23	39.53	5.63	0.10
**KNN**	7.83	7.83	0.08	7.76	7.75	0.08
**DAGMAM**	27.46	69.52	0.39	54.44	26.99	0.36
**LSTM-VAE**	96.24	59.91	0.74	87.79	14.45	0.25
**MAD-GAN**	98.97	63.74	0.77	41.44	33.92	0.37
**USAD**	98.51	66.18	0.79	99.47	13.18	0.23
USAD **	88.21	65.29	0.75	26.28 *	35.31 *	0.30 *
**CNN 1D**	94.25	67.92	0.78	39.30 *	20.28 *	0.27 *
**GDN**	99.35	68.12	0.81	97.50	40.19	0.57

**Table 5:** Anomaly detection accuracy (precision (%), recall (%), f1-score (%)) on two datasets after splitting into groups.

Method	SWAT			WADI *		
	Prec	Rec	f1	Prec	Rec	f1
**LSTM-VAE**	95.69	55.18	0.72	21.22	29.12	0.28
**USAD**	98.10	66.01	0.79	71.24	31.41	0.43
**CNN 1D**	95.24	63.73	0.78	63.76	43.54	0.52
CNN 1D ***	93.61	69.40	0.80	79.35	41.23	0.54

**Table 6:** CNN 1D model hyperparameters.

	Layer	Parameters		Layer	Parameters
1	Conv1D	in channels = 2 out channels = 50 kernel size = 4 padding = 1	7	Conv1D	in channels = 197 out channels = 131 kernel size = 5 padding = 1
2	Batch Norm1D	num features = 50	8	Batch Norm1D	num features = 131
3	Conv1D	in channels = 50 out channels = 131 kernel size = 2 padding = 1	9	Conv1D	in channels = 131 out channels = 50 kernel size = 2 padding = 1
4	Batch Norm1D	num features = 131	10	Batch Norm1D	num features = 50
5	Conv1D	in channels = 131 out channels = 197 kernel size = 5 padding = 1	11	Conv1D	in channels = 50 out channels = 2 kernel size = 4 padding = 1
6	Batch Norm1D	num features = 197	12	Batch Norm1D	num features = 2

## Data Availability

Restrictions apply to the availability of these data. Data was obtained from iTrust, Centre for Research in Cyber Security, Singapore University of Technology and Design and are available at iTrust with the permission of iTrust, Centre for Research in Cyber Security, Singapore University of Technology and Design.
